# Pro-inflammatory cytokines, but not brain- and extracellular matrix-derived proteins, are increased in the plasma following electrically induced kindling of seizures

**DOI:** 10.1007/s43440-020-00208-w

**Published:** 2020-12-30

**Authors:** Natalia Chmielewska, Piotr Maciejak, Bartosz Osuch, Miron B. Kursa, Janusz Szyndler

**Affiliations:** 1 Department of Neurochemistry, Institute of Psychiatry and Neurology, Sobieskiego Street 9, 02-957 Warsaw, Poland; 2 Interdisciplinary Centre for Mathematical and Computational Modelling, University of Warsaw, Pawinskiego Street 5A, 02-106 Warsaw, Poland; 3 Department of Experimental and Clinical Pharmacology, Centre for Preclinical Research and Technology CePT, Medical University of Warsaw, Banacha Street 1B, 02-097 Warsaw, Poland

**Keywords:** Hippocampal kindling, Peripheral inflammation, Seizure susceptibility biomarker, UCH-L1, MMP-9, GFAP

## Abstract

**Background:**

The aim of the study was to evaluate the brain-derived proteins, extracellular matrix-derived protein and cytokines as potential peripheral biomarkers of different susceptibility to seizure development in an animal model of epilepsy evoked by chronic focal electrical stimulation of the brain.

**Methods:**

The plasma levels of IL-1β (interleukin 1β), IL-6 (interleukin 6), UCH-L1 (ubiquitin C-terminal hydrolase 1), MMP-9 (matrix metalloproteinase 9), and GFAP (glial fibrillary acidic protein) were assessed. The peripheral concentrations of the selected proteins were analyzed according to the status of kindling and seizure severity parameters. In our study, increased concentrations of plasma IL-1β and IL-6 were observed in rats subjected to hippocampal kindling compared to sham-operated rats.

**Results:**

Animals that developed tonic–clonic seizures after the last stimulation had higher plasma concentrations of IL-1β and IL-6 than sham-operated rats and rats that did not develop seizure. Elevated levels of IL-1β and IL-6 were observed in rats that presented more severe seizures after the last five stimulations compared to sham-operated animals. A correlation between plasma IL-1β and IL-6 concentrations was also found. On the other hand, the plasma levels of the brain-derived proteins UCH-L1, MMP-9, and GFAP were unaffected by kindling status and seizure severity parameters.

**Conclusions:**

The plasma concentrations of IL-1β and IL-6 may have potential utility as peripheral biomarkers of immune system activation in the course of epilepsy and translational potential for future clinical use. Surprisingly, markers of cell and nerve ending damage (GFAP, UCH-L1 and MMP-9) may have limited utility.

## Introduction

Epilepsy is one of the most common neurological disorders and can be initiated by many genetic and nongenetic factors. The course, manifestation, and treatment response of epilepsy are not constant and may be modified by multiple endogenous and exogenous factors, such as age, sex, drugs, stage-specific effects, and comorbidities. The high level of complexity of epilepsy does not allow for the precise identification of basic target mechanisms for preventing or stopping epileptogenesis. Furthermore, the use of EEG for the diagnosis and treatment monitoring of epilepsy, as well as the differentiation of epilepsy from other disorders (i.e., from psychogenic seizures), may provide inconclusive results [1]. Thus, there is an urgent need to develop fast and noninvasive methods that allow the immediate diagnosis of seizures. In recent years, much attention has been paid to the detection of brain-derived proteins, which provide information about characteristic changes in epilepsy, such as blood–brain barrier (BBB) leakage, inflammation, gliosis, neuronal damage or death, and neuronal rearrangement. Additionally, some effort has been devoted to finding a correlation between the pattern of the peripheral release of these proteins and the severity of epilepsy symptoms. To date, none of the proposed blood biomarkers have become clinically relevant [2].

Many clinical and experimental findings suggest a link between immune system activation and the pathogenesis of epilepsy [3, 4]. However, it is unknown whether extensive immunological activation is a consequence of neuronal rearrangement during seizures or whether it is a causative factor linked to the pathogenesis of epilepsy [5]. The abnormal expression of cytokines and immune cells has been observed in key CNS areas linked to seizure generation in animal models. Cytokines, such as IL-1β and IL-6, have been revealed to be responsible for seizure and epileptogenesis propagation [6]. IL-1β has been shown to contribute to microglial activation, changes in astrocyte function, disruption of the BBB, and stimulation of other cytokine release. Similar functions can be assigned to IL-6. Some reports have indicated that inflammation may extend beyond the CNS, and increased levels of proinflammatory cytokines such as IL-1β and IL-6 have also been observed in the blood of epilepsy patients [2]. Under physiological conditions, cytokines have limited ability to cross the BBB. However, given the BBB disruption and extensive brain cytokine synthesis that may occur in epilepsy, it has been suggested that serum cytokine levels may reflect both CNS and peripheral production and may indicate immune activation [7]. Thus, there is convincing evidence for the potential of peripheral cytokines as a possible biomarker, and peripheral secretion may be an indicator of other significant epilepsy symptoms.

The other protein that has attracted significant interest as a potential biomarker for epilepsy, is ubiquitin C-terminal hydrolase (UCH-L1). It is estimated that UCH-L1 accounts for up to 5% of total neuronal protein [8]. It is a small 24-kDa neuron-specific cytoplasmatic enzyme that is required for the maintenance of axonal integrity under physiological conditions [8]. Uncontrolled expression of UCH-L1 has been described in the course of neurodegenerative diseases, such as Parkinson’s and Alzheimer’s disease. Furthermore, in some neurological conditions associated with the BBB breakdown and neuronal loss, UCH-L1 can permeate into the CSF and plasma [9]. Increased peripheral levels of UCH-L1 have been described after stroke, traumatic brain injury (TBI), and epilepsy, clinically and under experimental conditions [10–13]. The most advanced trial concerning the potential use of UCH-L1 as a biomarker of BBB disruption involved traumatic brain injury (TBI) [11]. It was found that the elevated level of UCH-L1 can predict positive CT scan results (signs of intracranial injury) [11]. However, these attempts seem insufficient, and more data are needed to support the use of this protein as an alternative to neuroimaging.

Matrix metalloproteinase 9 (MMP-9) is a proteolytic enzyme that rearranges the extracellular matrix (ECM) in physiological and pathophysiological processes [14]. MMP-9 cleaves the ECM also in the BBB by targeting tight junctions. MMP-9 is secreted from the dendrites of neurons following glutamate release [15]. According to recent studies, MMP-9 is thought to be strongly associated with the pathogenesis of many neurological disorders, such as Alzheimer’s disease, stroke, seizures and epilepsy [16, 17, 19]. It is mainly linked to impaired plasticity and excessive mossy fiber sprouting within the hippocampus, which in turn may have an impact on epileptic focus formation [18]. Moreover, MMP-9 may also contribute to increased inflammatory activity and BBB damage [19]. Preclinical studies have revealed that MMP-9 knockout mice develop seizures more slowly and that mice that overexpress MMP-9 are more prone to chemical kindling [20, 21]. In epilepsy patients, increased CSF and serum levels of MPP-9 have been found after tonic–clonic seizures and febrile seizures, probably due to increased BBB reorganization and leakage [22].

Glial fibrillary acidic protein (GFAP) is an astrocyte-specific protein that forms intermediate filaments in these cells. It plays a crucial role in maintaining the shape and motility of astrocytes. Furthermore, GFAP forms white matter structures and contributes to the BBB integrity. In addition, the expression of GFAP in hepatic stellate cells in normal livers was observed. The expression of GFAP increases in the course of neurological disorders associated with seizure generation, neurodegeneration, and brain injury [23–25]. Under physiological conditions, the peripheral concentration of GFAP is under the limit of detection or is at very low levels. Thus, an increase in GFAP concentration in the periphery may indicate a loss of astrocytic integrity and the disintegration of the BBB and may provide information about the severity of cellular changes in the CNS.

The purpose of this study was to evaluate certain brain-derived proteins and indicators of immune system activation as potential biomarkers of different susceptibility to seizure development. Furthermore, it was determined whether the peripheral levels of the chosen biomarkers correspond to seizure severity parameters. Thus, the plasma levels of the brain-derived proteins—UCH-L1, GFAP and ECM-derived protein—MMP-9, were measured. These proteins were chosen due to their confirmed role in the pathogenesis of epilepsy, as well as their ability to cross the BBB and stability in the bloodstream. Importantly, the selected markers represent different components of brain tissue: UCH-L1 indicates neurons; MMP-9 indicates ECM; and GFAP indicates astrocytes. Furthermore, markers of immunological activation, namely, IL-1β and IL-6, were also assessed. Due to the high interdependency of the processes in which the selected proteins are involved, correlations between protein concentrations was also assessed.

## Materials and methods

### Animals

Wistar male rats (*n* = 42 in total) weighing 250 ± 50 g at the beginning of the experiment were used. The animals were housed in standard laboratory conditions under a 12-h light/dark cycle at controlled temperature (20 ± 2 °C) and humidity (50%). The animals had free access to water and food. All experimental procedures were conducted between 9.00 am and 3.00 pm. The study was carried out in accordance with the European Communities Council Directive of November 24, 1986 (86/609/EEC) and was approved by the Committee for Animal Care and Use at the Medical University of Warsaw. All efforts were made to minimize suffering during the experimental procedures.

### Surgical electrode implantation

After 3 weeks of acclimatization, all rats (including sham rats) were implanted with a twisted bipolar electrode randomly into the left or right hippocampus. The coordinates for stereotaxic implantation were defined using the Paxinos and Watson Brain Atlas [26] and were as follows: ( −) 3.6 mm from bregma, 1.5 mm laterally, and 4 mm ventrally.

### Electrical kindling procedure

Two weeks after surgery, the rats (*n* = 28), excluding the sham-operated rats, which were electrode implantation controls (*n* = 14), were subjected to the electrical kindling procedure as described below. At the beginning of the procedure, the initial after-discharge threshold (AD) was measured. Stimulations were performed using a Grass Model S88 stimulator connected to a constant current unit (CCU1) and stimulus isolated unit (SiU5 RF; Grass Instruments; USA). A stimulus with a 1-s train of 60-Hz and 1-ms monophasic square-wave pulses at an intensity determined for each rat using AD measurements amplified by 25%, was delivered three times per week (Monday, Wednesday, and Friday, the same time each day). The animals were considered to be fully kindled after at least five consecutive stage 5 seizures were elicited (*n* = 8). Seizure stage was classified according to Racine’s scale: stage 1, jaw clonus; stage 2, head nodding; stage 3, forelimb clonus; stage 4, rearing on the hind limbs; and stage 5, loss of postural control and tonic–clonic seizures [27]. Rats that did not develop five consecutive stage 5 seizures were considered to be resistant (*n* = 20). We also decided to determine whether the levels of the plasma biomarkers correspond to the severity of the last seizure and the last five seizures. Based on the mean score of the last five seizures, we divided the rats into three groups: (1) Sham—sham-operated rats that were not electrically stimulated (*n* = 14); (2) Group “0–3.9”, which included rats with a mean seizure score of the last five stimulations of 0–3.9 (rats that exhibited partial seizure manifestation) (*n* = 12); and (3) Group “4.0–5.0”, which included rats with a mean seizure score of the last five stimulations of 4.0–5.0 (rats that mostly had tonic–clonic seizures during the last five stimulations) (*n* = 16). Based on the last seizure, the animals were divided into four groups: (1) Sham—which included sham-operated rats that were not electrically stimulated (*n* = 14); (2) Group “0”—which included animals that did not develop any seizure after last stimulation (*n* = 8); (3) Group “1–3”, which included animals that developed partial seizures after the last stimulation (seizure score from 1 to 3) (*n* = 4) and 4. Group “4–5”, which included rats that developed tonic–clonic seizures (seizure score from 4 to 5) after the last stimulation (*n* = 16).

### Determination of IL-1β, IL-6, UCH-L1, GFAP, and MMP-9 levels in the plasma

The animals were decapitated 24 h after the final AD examination, and the trunk blood was collected in heparinized tubes and centrifuged (2500×*g*, 15 min, 4 °C). The plasma was aliquoted and stored at − 80 °C until further analysis. After thawing, an extra centrifugation step was applied to prevent nonspecific signals from activated platelets (10,000×*g*, 10 min, 4 °C). The concentrations of IL-1β, IL-6, UCH-L1, GFAP and MMP-9 were measured using commercially available sandwich-based ELISA kits for rats (Elabscience, E-El-R0012 for IL-1β, detection range of 31.25–2000 pg/ml; Elabscience, E-El-R0015 for IL-6, detection range of 62.5–4000 pg/ml; R&D Systems, RMP900 for MMP-9, detection range of 0.156–10 ng/ml; MyBioSource Inc., MBS 2886354 for GFAP, detection range of 31.2–2000 pg/ml; Elabscience, for UCH-L1, detection range 61.25–4000 pg/ml). The optical density was determined using a microplate reader (SYNERGY H1, BioTek) set to 450 nm.

### Statistical analysis

According to the Shapiro–Wilk test, none of distributions of collected plasma concentrations of investigated components (IL-1β, IL-6, UCH-L1, GFAP, and MMP-9) is normal; to this end, non-parametric analysis methods are employed in this work. To compare data between kindled, resistant, and control animals, Kruskal–Wallis test followed by post hoc Conover-Iman test was used. For correlation analysis, Spearman’s coefficient was calculated. Statistical significance was defined as *p* < 0.05. Statistical analyses were performed using R (R Core Team, 2020) [28] with packages conover.test and pspearman.

## Results

### Changes in the plasma levels of IL-1β in rats after electrically induced kindling

**Fig. 1:**
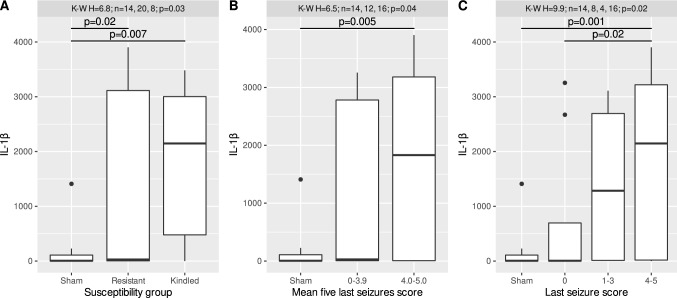
Plasma levels of IL-1β (pg/ml) in rats with respect to: **a** different susceptibility to electrical kindling, **b** mean five last seizures score and **c** last seizure score. Distributions of the data are shown in the form of Tukey’s boxplot; bold line denotes the median, while the box spans from the first to the third quartile. Whiskers denote the range of observations within 1.5 interquartile range from the box, while the remaining, extreme observations are marked with points. Sham—sham operated, not-kindled rats; resistant—rats, which has not gained criteria of kindling; kindled—rats which fulfilled kindling criteria. Annotations present *p* values of significant effects, established by the Kruskal–Wallis and Conover tests

Kruskal–Wallis test showed significant differences in IL-1β concentrations between rats with different kindling statuses (*H* = 6.8, *n*1 = 14, *n*2 = 20, *n*3 = 8, *p* = 0.03), between rats with different mean scores of the last five seizures (*H* = 6.5, *n*1 = 14, *n*2 = 12, *n*3 = 16, *p* = 0.04) and between rats with different scores of the last seizure (*H* = 9.9 *n*1 = 14, *n*2 = 8, *n*3 = 4, *n*4 = 16, *p* = 0.02). The Conover-Iman post hoc test revealed that IL-1β concentrations were significantly lower in sham-operated animals than either in animals susceptible to electrical kindling (*T* = 2.60, ν-39, *p* = 0.007) or in resistant animals (*T* = 2.09, ν-39, *p* = 0.02) (Fig. 1). Moreover, in the plasma of rats with mean seizure scores of the last five seizures of 4.0–5.0, the concentration of IL-1β was increased compared to that in the control rats (*T*-2.69, ν-39, *p* = 0.005). According to the last seizure score, rats that developed seizure scores of 4–5 exhibited a greater increase in IL-1β concentration than control rats and rats that did not develop seizures after the last stimulation (*T* = 3.25, ν-39, *p* = 0.001; *T* = 2.21, ν-39, *p* = 0.02) (Fig. 1).

### Changes in the plasma levels of IL-6 in rats after electrically induced kindling

**Fig. 2:**
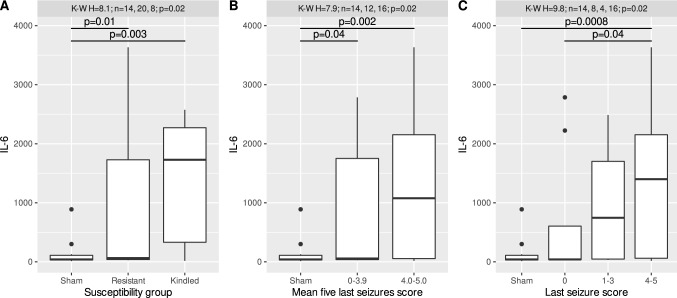
Plasma levels of IL-6 (pg/ml) in rats with respect to: **a** different susceptibility to electrical kindling, **b** mean five last seizures score and **c** last seizure score. Distributions of the data are shown in the form of Tukey’s boxplot; bold line denotes the median, while the box spans from the first to the third quartile. Whiskers denote the range of observations within 1.5 interquartile range from the box, while the remaining, extreme observations are marked with points. Sham—sham operated, not-kindled rats; resistant—rats, which has not gained criteria of kindling; kindled – rats which fulfilled kindling criteria. Annotations present *p* values of significant effects, established by the Kruskal–Wallis and Conover tests

Similarly to IL-1β, Kruskal–Wallis test revealed significant differences in IL-6 concentrations between control rats and both rats submitted to kindling and resistant ones (*H* = 8.1, *n*1 = 14, *n*2 = 20, *n*3 = 8, *p* = 0.02), between rats with different mean scores of the last five seizures (*H* = 7.9, *n*1 = 14, *n*2 = 12, *n*3 = 16, *p* = 0.02) and between rats with different scores of the last seizure (*H* = 9.8, *n*1 = 14, *n*2 = 8, *n*3 = 4, *n*4 = 16, *p* = 0.02). In a similar manner, post hoc test showed that IL-6 plasma levels were significantly higher both in animals submitted and resistant to electrical kindling than in sham-operated controls animals (*T* = 2.92, *ν* = 39, *p* = 0.003; *T* = 2.30, *ν* = 39, *p* = 0.01) (Fig. 2). Moreover, in the plasma of rats with mean seizure scores of the last 5 seizures of 0–3.9 and of 4.0–5.0 the concentration of IL-6 was increased compared to that in the control rats (*T* = 1.81, *ν* = 39, *p* = 0.04; *T* = 3.03, *ν* = 39, *p* = 0.02). In animals divided according to the last seizure score, rats with a seizure score of 4–5 exhibited a greater increase in the IL-6 concentration than both control rats and rats with a zero last seizure score (*T* = 3.39, *ν* = 38, *p* = 0.0008; *T* = 1.82, *ν* = 38, *p* = 0.04) (Fig. 2).

### Changes in the plasma levels of UCH-L1, MMP-9, GFAP in rats after electrically induced kindling

**Fig. 3:**
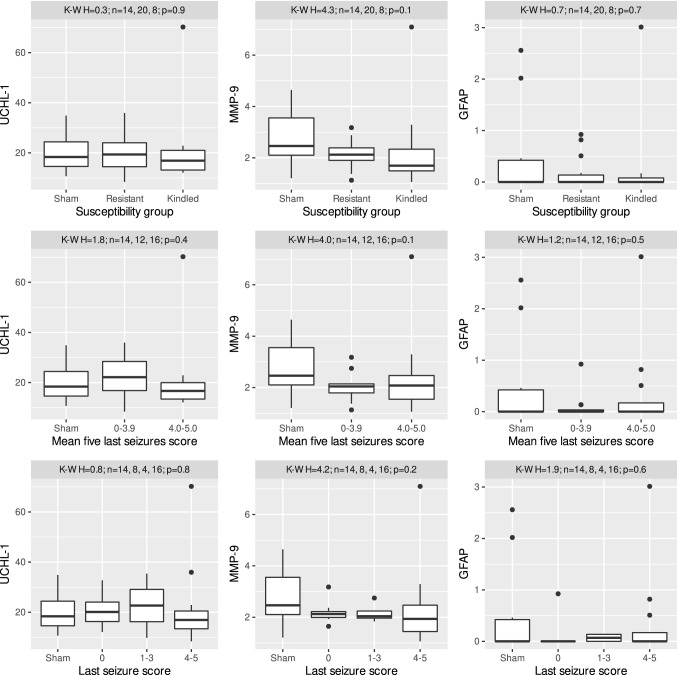
Changes in the plasma level of UCHL-1 (pg/ml; left panels), MMP-9 (ng/ml; center panels) and GFAP (pg/ml; right panels) according to: susceptibility group (top) mean five last seizures score (middle) and last seizure score (bottom). Distributions of the data are shown in the form of Tukey’s boxplot; bold line denotes the median, while the box spans from the first to the third quartile. Whiskers denote the range of observations within 1.5 interquartile range from the box, while the remaining, extreme observations are marked with points. Sham—sham operated, not-kindled rats; resistant—rats, which has not gained criteria of kindling; kindled—rats which fulfilled kindling criteria. None of the presented cases contained significant group differences, as established by the Kruskal–Wallis test, annotations present calculated *p* values

Kruskal–Wallis test did not reveal significant changes in the plasma levels of UCH-L1, MMP-9, or GFAP across experimental groups according susceptibility to kindling, the number of tonic–clonic seizures, mean seizure score during kindling, mean seizure score of the last five stimulations or score of the last seizure (Fig. 3).

### Correlation analysis

Spearman correlation analysis showed a significant correlation between IL-1β levels and susceptibility to kindling, treated as an ordered Sham < Resistant < Kindled feature (*ρ* = 41%, *n* = 42, *p* = 0.008), mean score of the last five seizures (*ρ* = 47%, *n* = 42, *p* = 0.002) and score of the last stimulation (*ρ* = 44%, *n* = 42, *p* = 0.004). Similar results were found for IL-6, which was also significantly correlated with susceptibility group (*ρ* = 44%, *n* = 42, *p* = 0.004), mean score over last five simulations (*ρ* = 47%, *n* = 42, *p* = 0.002), and score of the last stimulation (*ρ* = 44%, *n* = 42, *p* = 0.004). Correlation analysis between all plasma proteins measured revealed a correlation between IL-1β and IL-6 levels (*ρ* = 81%, *n* = 42, *p* < 0.001).

## Discussion

The purpose of the present study was to evaluate whether the peripheral levels of the brain-derived proteins UCH-L1 and GFAP, MCP-derived protein—MMP-9, and inflammatory cytokines, including IL-1β and IL-6, change in the course of kindling development and whether correspond to seizure severity parameters. In this study, we showed increased IL-1β and IL-6 plasma concentrations in animals that fulfilled the kindling criteria and in animals resistant to kindling development in comparison to control rats. Furthermore, we revealed a tendency for plasma IL-1β and IL-6 levels to increase in rats with higher mean scores of the last five episodes of seizures. Additionally, a higher concentration of IL-1β and IL-6 was observed in rats that developed tonic–clonic seizure after the last stimulation than in control rats. Correlations were found between plasma IL-1β and IL-6 concentrations, between IL-1β and IL-6 levels and susceptibility to kindling, and between IL-1β and IL-6 levels and the mean score of the last five episodes of seizures. On the other hand, the plasma levels of UCH-L1, MMP-9, and GFAP proteins were independent of kindling status, the mean score of the last five seizures and the score of the last seizure.

Under physiological conditions, IL-1β participates in the regulation of CNS functions. At low concentrations, IL-1β regulates core body temperature, modulates neuroendocrine responses, causes appetite suppression, induces slow-wave sleep, and contributes to synaptic plasticity processes [29]. On the other hand, IL-1β is considered a main proinflammatory factor and exhibits potential neurotoxic effects [30]. These effects include but are not limited to, neuronal loss, reactive astrogliosis, BBB leakage, and the activation of the local and peripheral inflammatory response associated with alternative activation of microglia/macrophages and other cytokines, such as IL-6 [30]. Focal or systemic inflammatory activation may cause aberrant hyperexcitability of neuronal transmission and alterations in connectivity, which in turn may contribute to the initiation of epileptogenesis [31].

CNS inflammation may be induced by local mechanisms or be acquired from the periphery, mostly due to disturbances in BBB function [4, 32, 33]. Furthermore, it contributes to the initiation, development, and persistence of epilepsy [3, 34, 35]. Preclinical data indicate increased hippocampal synthesis of IL-1β mRNA and protein in a number of experimental animal models of seizures and epilepsy induced by electrical or chemical triggers [36–39]. Human data also confirm the underlying inflammatory background of epilepsy [40]. Nevertheless, the presence and participation of inflammatory components in different types of epilepsy are not clear. An association between IL-1β gene polymorphisms and the risk of TLE (temporal lobe epilepsy) in individuals with TBI has been observed [41]. In patients with drug-resistant temporal lobe epilepsy (DRTLE), the neocortical levels of NFkB (nuclear factor kappa-light-chain-enhancer of activated B cells) and IL-6 were increased. The inflammation extended beyond the CNS, and the serum levels of IL-1 β and IL-6 were also increased. One year after the resection of epileptic foci in the same group of patients, the level of proinflammatory cytokines was decreased, and 70% of patients became seizure-free [41]. Furthermore, in some other clinical studies, the role of proinflammatory cytokines as potential biomarkers of epilepsy severity has been suggested [42–45]. Therefore, the American Epilepsy Society (AES) and the National Institute of Neurological Disorders and Stroke (NINDS) identified IL-1β as a potential biomarker of epilepsy [46].

In our previous study of pentylenetetrazole (PTZ)-induced seizures, a decreased concentration of IL-1β was found in rats resistant to seizures, and this change was linked to one of the mechanisms that may contribute to neuroprotection from seizures. These results are consistent with the literature and the data presented here. Inflammation in the course of epilepsy extends beyond the CNS and may be correlated with many clinical symptoms, such as risk of status epilepticus, drug resistance, and the frequency and severity of seizures. Our results confirm increased inflammation in rats subjected to kindling. However, even though many statistical and visual tendencies were observed, we did not find statistical significance for some seizure severity parameters. This was probably due to the strong interindividual variability in immune system activation in the course of epilepsy, which has also been observed clinically.

Recently, much attention has been devoted to the use of UCH-L1 as a biomarker of damage to neural cell bodies based on its neuron-specific expression within the CNS, low mass, and stability in the bloodstream. In our previous work PTZ kindled rats, we showed that 24 h after kindling episode the UCH-L1 concentration is increased in the plasma in all kindled groups of animals, regardless of different susceptibility to kindling. Therefore, we concluded that UCH-L1 is released after seizures and is an indicator of BBB leakage and enhanced neuronal loss in kindled animals compared to resistant rats rather than a marker of different susceptibility to kindling. Thus, we decided to include UCH-L1 in the panel of brain-derived proteins we studied in a model of electrically induced kindling. In the present study, plasma UCH-L1 levels did not differ between kindled, resistant and control animals. Furthermore, the peripheral level of UCHL-1 did not change based on the mean score of the last five seizures, the score of the last seizure, or susceptibility to kindling. These results are unexpected, not only because of our previous data but also because of the findings of other research groups, which identified UCH-L1 as a good indicator of seizure etiology, brain damage following seizures, and BBB leakage [11, 47, 48]. One of the possible explanations for this discrepancy may be that in electrically kindled animals, disturbances in the BBB function are not as strong and evident as in chemically induced seizures. In addition, the changes in the BBB are more centralized than in the case of chemical kindling, depended on the location of the stimulating electrode as well as on the average duration of the tonic–clonic seizure itself, which usually lasts shorter in electrical kindling. Such differences also may be due to the specificity of electrical kindling, in which EEG recordings allow to adjust the parameters of stimulation individually for each rat according to its susceptibility to developing afterdischarges. In the chemically induced model, the dose of PTZ is constant for each rat independent of susceptibility to seizures. Finally, human studies enroll patients with long-lasting, chronic disease manifestations.

MMP-9 participates in many physiological and pathological processes. In the nervous system, its function is associated with neural rearrangement, synaptic reorganization, and participation in remyelination [49]. Additionally, a role for MMP-9 in the regulation of BBB permeability due to the degradation of ECM as well as the opening of the tight junctions has also been indicated [50]. In MMP-9 knockout mice, diminished sensitivity to kindling has been reported, and inversely, mice that overexpress MMP-9 have increased susceptibility to kindling [18]. Similarly, in our previous work, we showed a decrease in the peripheral concentration of MMP-9 in rats resistant to chemical kindling, and we related this to a lower propensity for kindling [10]. Here, we did not observe changes in plasma MMP-9 concentration between rats with different susceptibility to kindling or between rats according seizure severity parameters. It seems likely that the role of MMP-9 in electrically induced kindling may be restricted, in this specific case, to the epileptic focus, especially, when we consider data showing that MMP-9 is strongly associated with the formation of a new epileptic focus in TLE epilepsy as a prime activator and deactivator of many chemokines and cytokines [51].

In previous studies of PTZ-induced kindling in rats and in the current study, we did not observe changes in the plasma GFAP concentration based on different susceptibility to kindling or other seizure severity parameters. Similarly, in only 3 of 29 patients with epilepsy, the peripheral GFAP level was shown to be increased after status epilepticus [52]. Additionally, in 7 of 52 children, the CSF GFAP level was elevated 24 h after seizure [53]. Our preclinical studies and other clinical studies imply that GFAP is not a reliable peripheral marker in epilepsy. This is probably because astrocyte injury rarely occurs under these condition, and further, the pivotal role of astrocytes in the formation of epileptic lesions and gliosis may indicate exclusive expression of GFAP in the CNS.

## Conclusion

From the clinical point of view, looking for peripheral biomarkers, that could predict the onset of epilepsy, following different insults such as brain injury, may enable the early introduction of preventive treatment for epilepsy. In addition, excessive immunological activation may be responsible for the prominent mechanism of seizure and diminished responsiveness to the antiepileptic drugs.

To date, despite many years of research, the one, specific form epilepsy, peripheral biomarker has not been identified. At present, because of the high complexity of the aetiology of epilepsy, it seems that more useful to this end would be panels of the peripheral biomarkers, specific for epilepsy. In our study, peripheral concentrations of IL-1β and IL-6 corresponded to kindling and seizure severity parameters. On the other hand, it was demonstrated that the brain-derived proteins UCH-L1, GFAP and ECM-derived—MMP-9 are not released into the periphery in a manner that is dependent on seizure severity or kindling and failed to show their potential as peripheral biomarkers.

In that context, we believe that the novel potential biomarkers should not stand alone as a crucial decision making factor. New therapeutic strategies need to integrate clinical information coming from medical history, EEG and neuroimaging with information coming from peripheral biomarkers assessment.
